# Reliability and Recommended Settings for Pediatric Circumpapillary Retinal Nerve Fiber Layer Imaging Using Hand-Held Optical Coherence Tomography

**DOI:** 10.1167/tvst.9.7.43

**Published:** 2020-06-30

**Authors:** Sonal D. Shah, Adnaan Haq, Shafak Toufeeq, Zhanhan Tu, Budor Edawaji, Joseph Abbott, Irene Gottlob, Frank A. Proudlock

**Affiliations:** 1 University of Leicester Ulverscroft Eye Unit, Department of Neuroscience, Psychology and Behaviour, University of Leicester, Robert Kilpatrick Clinical Sciences Building, Leicester Royal Infirmary, Leicester, UK; 2 Moorfields Eye Hospital NHS Foundation Trust, London, UK; 3 Ophthalmology Department, Stoke Mandeville Hospital, Aylesbury, UK; 4 Ophthalmology Department, Birmingham Children's Hospital, Birmingham, UK

**Keywords:** OCT, pediatrics, retinal nerve fiber layer, reliability

## Abstract

**Purpose:**

To investigate feasibility and reliability of 3-dimensional full circumpapillary retinal nerve fiber layer (cpRNFL) analysis in children, with and without glaucoma, without the use of sedation and to recommend a protocol for hand-held optical coherence tomography use.

**Methods:**

A cohort of pediatric glaucoma patients and normal children were imaged with hand-held optical coherence tomography to assess the feasibility of obtaining full cpRNFL. Two consecutive scans were acquired in a smaller sample to investigate test–retest repeatability and interassessor reproducibility. The cpRNFL thickness was assessed in four quadrants, at several visual angles from the optic nerve center.

**Results:**

Scanning was attempted in both eyes of 90 children with pediatric glaucoma and 180 controls to investigate feasibility (mean age, 6.98 ± 4.42 years). Scanning was not possible in 68 eyes of glaucoma children mainly owing to nystagmus, unclear optical media, or high refractive errors. Where three-dimensional imaging was possible, success at obtaining full cpRNFL was 67% in children with glaucoma and 89% for controls. Seventeen children with pediatric glaucoma and 34 controls contributed to reliability analysis (mean age, 6.3 ± 3.63 years). For repeatability intraclass correlation coefficients across quadrants ranged from 0.63 to 0.82 at 4° and improved to 0.88 to 0.94 at 6°. Intraclass correlation coefficients for reproducibility were also highest at 6° (>0.97 across all quadrants).

**Conclusions:**

We demonstrate that acquisition and measurement of cpRNFL thickness values using 3-dimensional hand-held optical coherence tomography volumes in awake children is both feasible and reliable and is optimal at 6° from optic nerve center.

**Translational Relevance:**

Our recommended protocol provides guidance on how pediatric optic nerve pathologies are managed by clinicians.

## Introduction

Optical coherence tomography (OCT) imaging has revolutionized the diagnosis and management of adult eye diseases in ophthalmology.^1^ Three-dimensional imaging of the circumpapillary retinal nerve fiber layer (cpRNFL) has been an important diagnostic marker, especially for detecting cpRNFL thinning caused by glaucoma.^2^^,^^3^ CpRNFL thinning also occurs in conditions such as multiple sclerosis^4^ and Alzheimer's disease.^5^ However, in infants and younger children, lack of cooperation has hindered acquisition of 3-dimensional images. Hand-held OCT (HH-OCT) has recently made acquisition of OCT images in infants and young children much easier, advancing the understanding of pediatric ocular development.

HH-OCT has been used to map foveal and optic nerve development in normal young children,^6^ and also in preterm infants.^7^^,^^8^ It has also been shown to be reliable in children with infantile nystagmus.^9^ More recently, abnormalities of the RNFL and optic nerve have been shown in congenital glaucoma.^10^ However, these studies mainly analyzed single central B-scans through the fovea or optic nerve. Comparison of interexaminer analyses of single B-scan images find reproducibility to be excellent for optic nerve diametric parameters from children aged 0 to 13 years,^11^ although the reproducibility of RNFL thickness is lower.

Acquiring a scan of the entire optic nerve region is more difficult in children and automatic segmentation is not widely available. The advantage of volumetric imaging of the optic nerve region over single B-scan analysis, however, is that it captures arcuate retinal nerve fiber bundles in the superior and inferior quadrants,^12^^,^^13^ often most susceptible to damage in optic nerve pathologies. It is not clear how feasible and reliable acquisition and analysis of the entire cpRNFL is in awake children. To date, full cpRNFL imaging using HH-OCT is limited to children under anaesthesia.^14^ Avery et al*.*^14^ demonstrated good intervisit and intravisit reproducibility of cpRNFL thickness measures in sedated children with optic pathway gliomas. Rothman et al*.*^15^^,^^16^ have also described high intergrader and intragrader and intravisit reproducibility for partial circumferential RNFL analysis across the temporal sectors in preterm and full-term babies, aged 36 weeks or less, or aged between 37 and 42 weeks, respectively. They investigated RNFL measurements at various radii from the optic nerve center but only describe intergrader and intragrader and intravisit reproducibility for one radius (1.5 mm) for one-half of the cpRNFL.

We investigate the reliability of full circumferential RNFL analysis in infants and children, with and without glaucoma, without the use of sedation using HH-OCT, assessing repeatability (test–retest reliability) and reproducibility (interassessor reliability). We also assess the reliability of different quadrants of the cpRNFL, and investigate the effect of the measuring distance from the optic nerve center, comparing annular and ring measures. Our aim is to develop optimized settings for cpRNFL acquisition in infants and children using the Leica HH-OCT system.

## Methods

### Subjects

Children were consecutively enrolled to the study from pediatric glaucoma clinics at Birmingham Children's Hospital, UK if they had either primary or secondary glaucoma. At the beginning of a clinic, patient notes were reviewed to identify potential participants. Patients underwent an orthoptic assessment followed by an ophthalmologic examination as a part of their clinical assessment, which was used to establish if they met inclusion criteria. Healthy children were recruited from local schools and nurseries in Leicestershire and delivery suites at the Leicester Royal Infirmary. Informed written consent was obtained from all participants and assent from children if appropriate.

Children were diagnosed as having glaucoma based on the definitions by the Childhood Glaucoma Research Network, in the 9th Consensus Report of the World Glaucoma Association (www.worldglaucoma.org). The inclusion criteria for control participants included best-corrected visual acuity appropriate for age, and refractive error within ±3 diopters; exclusions were any systemic or neuro-ophthalmic conditions, and birth gestation of less than 37 weeks.

The study adhered to the tenets of the Declaration of Helsinki, and Ethics Committee approval was obtained.

### HH-OCT Image Acquisition

All participants were imaged using the HH-OCT device (Leica Microsystems, Envisu C2300, Wetzlar, Germany; axial resolution of 2.4 µm). Volumetric images were acquired using a 12 mm x 8 mm horizontal raster scan protocol (600 A-scans x 80 B-scan, adult settings before correction for axial length), which was based on the following factors:

• A rectangular scan of 12 mm x 8 mm allowed the fovea and cpRNFL region to be acquired in the same scan with a high level of success (Tables 1 and 2). A ring scan was not feasible because of difficulties in locating the center of the scan at the optic disc center.
• An A-scan spacing of 20 µm was sufficient to visualize the RNFL on a single B-scan.
• A B-scan spacing of 100 µm was sufficient to visualize the optic nerve head (approximately 10–15 scans to resolve the disc vertically).
• The Leica system ‘Free run’ continuous real-time imaging mode refreshing every 1.68 seconds, which gave the user sufficient time to stop the acquisition to keep the last acquired image.

**Table 1.** Feasibility of Capturing Full cpRNFL Thickness

	Glaucoma	Controls
	Eyes (Children)	Eyes (Children)
Summary of number of scanning attempts for children included in the feasibility analysis		
Number of eyes where scanning was attempted	256 (90)	360 (180)
Number of eyes where 3D imaging was possible	188 (64)	360 (180)
Number of eyes where 3D imaging was not possible	68 (31)	0 (0)
Factors preventing acquisition of 3D image		
Motion owing to nystagmus	24 (10)	0 (0)
Cloudy or opaque optical media	30 (12)	0 (0)
High uncorrected refractive error	10 (6)	0 (0)
Very small pupils	2 (1)	0 (0)
Other	2 (2)	0 (0)
Total	68 (31)	0 (0)

3D, three-dimensional.

**Table 2.** Feasibility Analysis for Each Individual Quadrant and Full cpRNFL

		Success Rate (%)				
		cpRNFL Quadrant				
Participant Group	*n*	Nasal	Temporal	Inferior	Superior	Full
Glaucoma						
From all eyes where 3D imaging was possible	188	85.1	88.8	82.4	77.1	67.0
From all eyes where scanning was attempted	256	62.5	65.2	60.5	56.6	49.2
Controls						
From all eyes where 3D imaging was possible	360	97.2	98.6	92.8	93.6	88.9
From all eyes where scanning was attempted	360	97.2	98.6	92.8	93.6	88.9
Total						
From all eyes where 3D imaging was possible	548	93.1	95.3	89.2	88.0	81.4
From all eyes where scanning was attempted	616	82.8	84.7	79.4	78.2	72.4

3D, three-dimensional.

OCT scans were performed in an outpatient setting, without the use of anesthetics or dilation drops in children. Neonates and infants were supine in a cot or parents’ arms, whereas older children sat upright with fixation targets such as toys and cartoon videos used for distraction. The OCT lens was held by the examiner between the index finger and thumb and motion of the probe stabilized relative to the child's eye by placing the other fingers against the forehead of the patient. This maneuver also provides fine control of the position of the lens relative to the eye. Choosing the Free run mode allowed real-time imaging on the visual display unit, so the examiner can position the lens to include both the optic nerve and fovea. The examiner captured the scan by communicating with the second assisting examiner. The volume was reviewed by the examiner and saved if considered sufficient, or another attempt was made.

Each patient had two consecutive scans taken on the same day, in the same eye, by the same examiner in one affected eye. Because glaucoma is a dynamically changing condition, repeated measures were recorded in the same imaging session. The matching eyes of controls also had two consecutive scans taken in the same session. OCT scans were included in reliability analysis when at the least three cpRNFL quadrants were captured in the two consecutive scans (same three quadrants). OCT scans containing motion artefacts were not accepted. Using the Free run mode allowed the examiner to save the optimal OCT volume. The imaging probe was repositioned between the two consecutive scans.

For the feasibility analysis, each participant required a single scan taken in each eye on a visit. We retrospectively gathered all patients with glaucoma.

### HH-OCT Analysis

Images were converted using an ImageJ (http://imagej.nih.gov/ij/; provided in the public domain by the National Institutes of Health, Bethesda, MD)^17^ script into a format that could be imported into Copernicus SR analysis software (Optopol Technology, Zawiercie, Poland). The image was cropped down to an 8 mm x 8 mm window (adult settings before correction for axial length) centered on the optic nerve head (ie, 600 A-scans width was cropped to 400 A-scans width). The automatic segmentation was manually inspected by the examiner to correct any segmentation errors.

**Figure 1.**
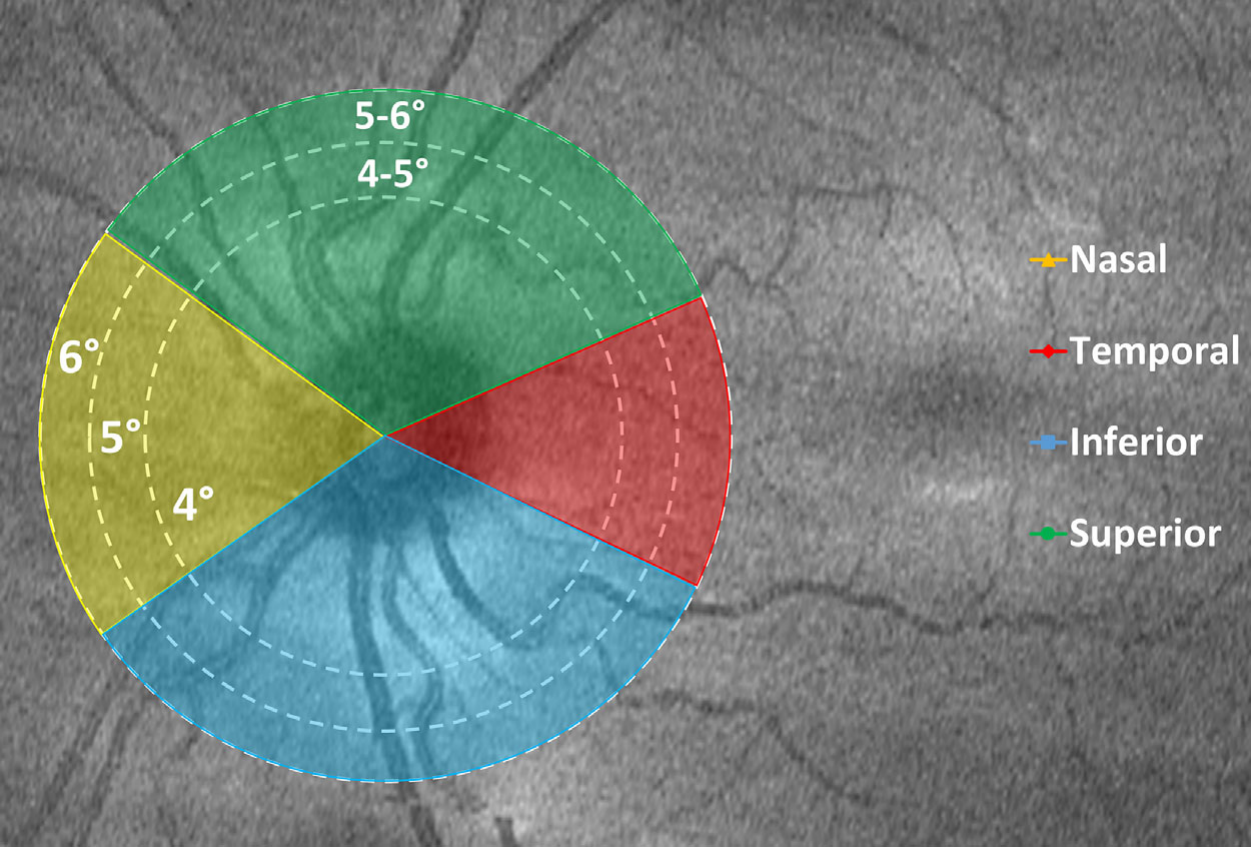
The cpRNFL quadrants, and circular ring and annuli. A color-coded schematic to illustrate each quadrant and their proportions; circular rings of 4°, 5°, and 6° visual angles from the optic nerve center; and circular annuli of 4° to 5° and 5° to 6°.

The cpRNFL thickness was assessed at three circular rings from the optic disc center at 4°, 5°, and 6° radii; and two annuli between 4° and 5° and 5° and 6°; and for nasal, temporal, inferior, and superior quadrants (using GDx Nerve Fiber Analyzer protocol, Carl Zeiss Meditec) as illustrated in Figure 1. A conversion factor provided by Leica Microsystems was used to convert lateral distances to visual angles (288 µm/degree in adult eyes). In a recent study^11^ characterizing the time course of development of the optic nerve head region in the early years of life using HH-OCT, we find that parameters such as optic disc and cup width remain constant with age when expressed as a visual angle rather than as a lateral distance measurement. We suggest this is because these parameters increase proportionally with increasing axial length and that the expansion of the scleral shell is an important factor determining growth of the cup and disc in the first 2 years of life.

### Feasibility

Feasibility was investigated for each separate quadrant, and the success of obtaining a full cpRNFL scan. Factors preventing acquisition of three-dimensional imaging were explored.

### Reliability Analysis

The consistency of the automatic segmentation with manual correction of images recorded from children with and without glaucoma were evaluated for HH-OCT images recorded in the same session, from the same eye by the same assessor.

#### Repeatability (Test–Retest Reliability)

The two consecutive scans, scan 1 and scan 2, from each participant were categorized as high quality or low quality, respectively, using the quality index provided by the software. The quality index is based on the image contrast between vitreous and retinal tissue. This approach allowed us to determine whether image quality introduced a bias in measurements.

#### Reproducibility (Interassessor Reliability)

The two consecutive scans taken by one examiner were segmented by two assessors, assessor 1 and assessor 2, and compared.

### Statistical Analysis

Statistical analysis was carried out using SPSS software version 24 (SPSS, Inc., Chicago, IL) and Microsoft Excel 2013. The Shapiro-Wilk test was used to determine normality of cpRNFL thickness distributions; allowing parametric statistical tests to be used. Interdevice reliability, test–retest reliability, and interassessor reliability were evaluated using the intraclass correlation coefficient (ICC) and the coefficient of variation (CoV). Biases were calculated (with 95% confidence intervals) and assessed for statistical significance using linear mixed models including eye and assessor as fixed factors.

Bland-Altman plots have been used to highlight reliability agreement at varying visual angles from the optic nerve center.

## Results

### Participants

#### Feasibility

Ninety children with pediatric glaucoma were recruited (mean age, 6.98 ± 4.42 years; range, 0.05–16.71 years). OCT scans were also attempted on children attending follow-up clinical visits, regardless of the success on their first visit, resulting in 256 scans from 90 children. From the 90 children, 10 had nystagmus, 12 had buphthalmos or other corneal opacities, 6 had uncorrected high refractive error, 1 had very small pupils, 1 had a phthisical eye, and 1 child had a shield over 1 eye after surgical intervention. There were 180 age-, gender-, and ethnicity- matched controls (ratio 1:2, glaucoma:controls) (mean age, 7.09 ± 4.56 years; range, 0.02–16.49 years) who were included in this analysis. Table 1 summarizes the number of scanning attempts for children included in the feasibility analysis.

#### Reliability

Seventeen affected eyes from 17 pediatric glaucoma patients were included in test–retest and interassessor reliability analysis. The age of children (mean age, 6.3 ± 3.63 years; range, 0.8–14.7 years) represented those attending the glaucoma clinics. There were 34 age-, gender-, and ethnicity-matched controls (ratio 1:2, glaucoma:controls) (mean age, 6.1 ± 3.67 years; range, 0.8–15.4 years) who were included in this analysis.

A sample size of at least 16 children with glaucoma was based on the study by Schrems-Hoesl et al.^18^ A ratio of 2:1 controls:glaucoma children was used because a large sample of normative age-matched controls was available.

From the two consecutive scans attempts, the first scan for each patient and control captured all four quadrants. Four of 17 patients and 2 of 34 controls had one quadrant missing in the second scan.

**Figure 2.**
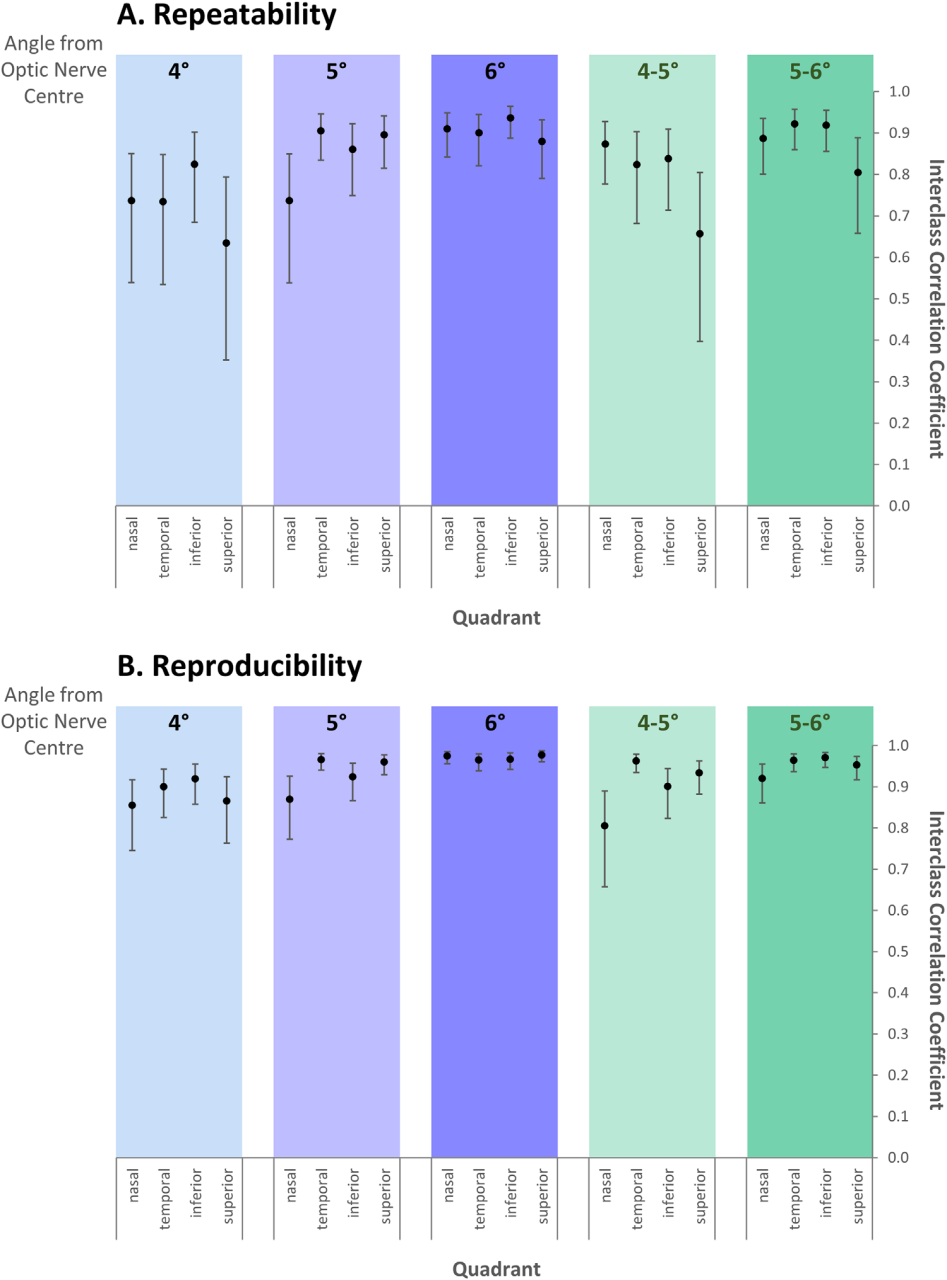
ICC. Graphs to illustrate ICC at varying distances from the optic nerve center for measures of (A) repeatability (test–retest reliability) and (B) reproducibility (interassessor reliability). Error bars represent upper and lower 95% confidence intervals.

Reliability improved further away from the optic nerve center, across all quadrants, evident from the smaller biases, smaller coefficients of variation, and higher interclass correlation coefficients with increasing distance from the optic nerve (Fig. 2, Tables 3 and 4, and Supplementary Tables S1 and S2) for repeatability and reproducibility. Measurements at 6° from the optic nerve center generated the most reliable measurements.

**Table 3.** Repeatability Variables (Test-Retest Reliability) for the Circumpapillary Measurements at 6° from the Optic Nerve Centre

	Mean ± SD RNFL Thickness (µm)		Bias (µm)				Reliability			
Quadrant (n)	High Quality	Low Quality	Mean	*P* Value	LCI	UCI	CoV (%)	ICC	LCI	UCI
Nasal (*n* = 51)	65.44 ± 12.49	64.92 ± 12.02	0.52	0.44	−0.80	1.84	6.05	0.88	0.79	0.93
Temporal (*n* = 51)	61.12 ± 8.42	60.05 ± 8.43	1.07	0.11	0.22	1.92	4.08	0.91	0.84	0.95
Inferior (*n* = 49)	104.64 ± 16.08	105.55 ± 15.59	−0.96	0.25	−2.62	0.69	4.67	0.90	0.82	0.94
Superior (*n* = 47)	106.43 ± 15.52	106.35 ± 16.40	0.06	0.93	−1.32	1.45	3.81	0.94	0.89	0.96
Average (*n* = 45)	90.57 ± 12.57	92.39 ± 11.47	0.14	0.77	−0.79	1.06	9.03	0.95	0.90	0.97

Mean and standard deviation of hand-held OCT is provided along with the bias with upper and lower 95% confidence intervals (LCI and UCI) for repeatability (test–retest reliability). Reliability is assessed using CoV, and ICC with upper and lower 95% confidence intervals.

The sample size for the repeatability analysis was determined by the number of low quality scans available (equivalent to the *n* values). Only participants who had data for all four quadrants for both scans were included in the average measure analysis.

**Table 4.** Reproducibility Variables (Interassessor Reliability) for the Circumpapillary Measurements at 6° from the Optic Nerve Centre

	Mean ± SD RNFL Thickness (µm)		Bias (µm)				Reliability			
Quadrant (n)	Assessor 1	Assessor 2	Mean	*P* Value	LCI	UCI	CoV (%)	ICC	LCI	UCI
Nasal (*n* = 51)	65.37 ± 12.24	64.99 ± 12.28	0.38	0.57	−0.94	1.70	1.95	0.98	0.96	0.99
Temporal (*n* = 51)	60.59 ± 8.42	60.58 ± 8.43	0.01	0.98	−0.84	0.86	1.91	0.97	0.94	0.98
Inferior (*n* = 51)	104.54 ± 15.83	104.86 ± 15.83	−0.34	0.68	−1.96	1.28	2.00	0.97	0.94	0.98
Superior (*n* = 51)	106.22 ± 15.57	106.15 ± 16.47	0.07	0.92	−1.31	1.45	1.75	0.98	0.96	0.99
Average (*n* = 51)	89.04 ± 14.76	89.05 ± 15.21	−0.01	0.99	−1.65	1.63	2.46	0.99	0.98	0.99

Mean and standard deviation of hand-held OCT is provided along with the bias with upper and lower 95% confidence intervals (LCI and UCI) for reproducibility (interassessor reliability). Reliability is assessed using CoV, and ICC with upper and lower 95% confidence intervals.

As expected, RNFL thickness values for the control group were greater than those in the glaucoma group at all distances from the optic nerve center as seen from the distribution of the points of the Bland-Altman plots (glaucoma in blue and controls in red) along the *x*-axes in supplementary Figures 1A and B. For Bland-Altman plots, there was no significant trend in differences (either high quality – low quality or assessor 1 – assessor 2) with the mean value for any quadrant or location away from the optic nerve (*P* > 0.5).

#### Repeatability: Test–Retest Reliability

The ICCs for the test–retest reliability improved from 0.63 to 0.82 at 4°, to 0.88 to 0.94 at 6° (Fig. 2A and Supplementary Table S2). At 4° the bias was small (range, –1.94 to 5.91 µm), whereas the CoV was higher (range, 9.15%–11.36%); the nasal and superior quadrants showing the worst results. All ICC results remained the same or improved at a further distance from the optic nerve center, and lower and upper 95% confidence intervals became smaller (Fig. 2A). In Supplementary Figure S1A, the wider spread of points in the Bland-Altman plot below compared with above 0 µm reflects the effect of poor quality scans. Improvement in the variation of results from 4° to 6° in the nasal quadrant can also be seen. The RNFL thickness for the temporal quadrant measured at 5° was significantly thinner for the lower quality scan (*P* = 0.022), although reliability was still high (CoV, 4.75%; ICC, 0.91).

#### Reproducibility: Interassessor Reliability

The RNFL data from the same cohort of children included for repeatability was segmented by assessor 1 and assessor 2 to measure interassessor reliability. The ICCs at 4° ranged from 0.86 to 0.92 (Fig. 2B and Supplementary Table S1), with biases ranging from –6.44 to 3.69 µm; again, the nasal and superior quadrants showed the worst results. This finding is illustrated by the Bland-Altman plots in Supplementary Figure S2B. The highest reproducibility was at 6° with ICCs of greater than 0.97 across all quadrants, with a small bias (range, –0.34 to 0.38µm) and low CoV (range, 1.75%–2.00%).

## Discussion

In this study, we investigated repeatability (test–retest reliability) and reproducibility (interassessor reliability) of full cpRNFL in infants, using a semiautomated technique. We found the most reliable results were obtained at a radius of 6° visual angle from the optic nerve center. High ICCs for repeatability and reproducibility were achieved in all quadrants at 6°, 0.88 to 0.94 and 0.97 to 0.98, respectively (Table 3 and Table 4), accompanied with small bias and CoV values. An annulus of 5° to 6° also demonstrated high reliability.

### Feasibility

The feasibility analysis indicated that factors associated with the pathology of glaucoma, such as nystagmus, cloudy or opaque optical media, or high uncorrected refractive error, were a limiting factor bringing feasibility to 65% or lower for most quadrants. Where three-dimensional imaging was possible, the feasibility of individual quadrants was between 77% and 89% in glaucoma, suggesting that the challenges of carrying out HH-OCT are not a significant limiting factor in this group of children.

### Repeatability and Reproducibility

Several studies report normative RNFL thickness values for older children using time-domain and spectral-domain table-mounted OCT devices,^19^^,^^20^ with high reliability of results,^21^ comparable with those from adults. Altemir et al*.*^21^ report ICCs for intraobserver repeatability of RNFL thickness measures at a distance of 1.73 mm in normal children aged 6–11 years using spectral-domain OCT, as ranging from 0.88 to 0.93 and CoVs ranging from 3.93% to 5.52%. Interobserver reproducibility ICCs ranged from 0.88 to 0.93 and CoVs from 3.41% to 5.18%. Avery et al*.*^14^ also demonstrated good reliability of cpRNFL thickness values using HH-OCT in sedated younger children, aged 0.8 to 13.0 years, affected with optic gliomas. Reliability was mostly unaffected by whether an isotropic (300 x 300) or nonisotropic (1000 x 100) scan was used. They found intravisit repeatability ICCs ranging from 0.93 to 0.99, with CoVs ranging from 1.50% to 11.5%. In our study the repeatability ICCs at 6° were 0.88 to 0.94, with CoVs of 3.81% to 6.05%. Thus, using sedation improves repeatability probably because of reduced motion artefacts. Without use of sedation, the use of fixation targets is a key element in keeping young children distracted, combined with the Free run mode on the Leica HH-OCT device to make image acquisition a rapid process.

With respect to repeatability in younger children Rothman *et al.*^15^ reported ICCs ranging from 0.37 to 0.92 in newborns, although the reliability of different quadrants was not reported. They also report intergrader reproducibility with ICCs ranging from 0.75 to 0.95, whereas we reported higher ICCs between 0.97 and 0.98. The more variable findings reported by Rothman et al.^15^ may be due to the small sample size used (*n* = 12), the young age group, or possibly the different segmentation method used. Patel et al.^11^ also report ICCs ranging from 0.77 to 0.68 for interexaminer reproducibility of point measures on temporal and nasal aspects (6°) of the optic nerve in a large cohort of 218 normal children aged 0 to 13 years. These were lower than ICCs for our quadrant measures (>0.97).

In general, interassessor reproducibility was better than test–retest reliability. This finding indicates that factors such as repositioning the image probe between scans, and small-scale motion artefacts (scans with obvious motion artefacts were excluded) owing to refixations and hand movements introduces a degree of variability between measurements. However, ICCs between 0.88 and 0.94 at 6° from the optic nerve center provide evidence that reliable measures are possible despite these factors.

### Optimal Scan Radius for cpRNFL Measurements

#### Distance

In table-mounted OCT devices, typically a fixed scan radius of 1.70 to 1.75 mm from the optic nerve center is used for RNFL measurements, ensuring there is no overlap of the optic nerve head whilst providing sufficiently thick RNFL to detect any subtle changes.^22^ Rothman et al.^15^ have assessed the temporal RNFL at 1.1, 1.3, 1.5, and 1.7 mm radii from the optic nerve center in healthy full-term neonates. They report a radius of 1.5 mm (equivalent to 5.2° on Leica the HH-OCT system) as optimal based on this location generating the most normally distributed data. In our study, we assess the validity, reproducibility, and repeatability and demonstrate that a visual angle of 6° from the optic nerve center is optimal for all three reliability measures.

#### Visual Angles

Patel et al.^11^ have explored the difference between defining lateral measures as visual angles compared with lateral distances (6° compared with 1.7 mm from the center of the nerve, respectively) on optic nerve and cpRNFL parameters measured in children from birth to 13 years of age. When measured at 1.7 mm, the temporal RNFL thickness initially decreased from birth to 18 months, followed by a slow increase in thickness until 13 years of age. However, at a 6° radius after the initial thickness decrease the RNFL thickness remained constant. Patel et al.^11^ argue that these differences illustrate that defining lateral measures as visual angle is advantageous as it reduces the error of not adequately correcting for age-related axial length changes.

We, therefore, recommend using a fixed visual angle of 6° across all age categories to measure cpRNFL. A measurement at 6° is also less affected by superficial vasculature which is conventionally included in cpRNFL segmentation analysis and is more dispersed away from the optic nerve.

### Limitations

There are several potential limitations in this study. The difficulty to keep infants fixating straight ahead with good head positioning is inevitable. Because head tilt causes the optic disc to appear rotated on OCT scans,^23^ the quadrants were not always representative of the same area of RNFL being measured, resulting in variability of RNFL thickness measures between two scans from one individual. However, this represents the real life clinical situation on how the scans are acquired. A future improvement could be made by using the fovea to optic nerve center plane as a reference to define cpRNFL quadrants.

Hwang et al.^23^ investigated the effects of horizontal head tilt on RNFL thickness values in adult participants. Induced optic disc rotation clockwise and counterclockwise of 8.27° and 8.47°, respectively, resulted in small but significant RNFL thickness changes in the nasal and temporal quadrants, ranging from 1.60 to 2.67 µm. Owing to a lack of an internal target, children imaged with the handheld device were also prone to convex or concave-tilted OCT scans, caused by an off pupil-centered OCT beam.^24^ Tilted scans often result in artefactual thickening or thinning of RNFL measures.

In addition to head tilt, probe misalignment away from the sagittal plane can also result from the manual alignment of the probe with the child's head by the assessor. In adults, the use of a head and chin support minimizes this artifact.

Interassessor reproducibility was a measure of the consistency of manual correction following automated segmentation between two assessors. There are other sources of variability that have not been assessed in this study that could be caused by a child attending repeat visits, such as images being acquired by different assessors, in a different clinical setting and using a different imaging device or protocol.

## Summary

We have demonstrated that it is feasible to obtain complete cpRNFL in nonsedated children using HH-OCT, generating reliable cpRNFL thickness values, for three-dimensional analysis. Table 5 summarizes recommended protocol settings for pediatric imaging using Leica HH-OCT. Although OCT has become a fundamental tool in the management of glaucoma and other optic nerve pathology in adults,^25^ to date infants with congenital glaucoma and other optic nerve pathologies have largely had these quantified, structurally, by en face ophthalmoscopic appearances.^26^ Based on our findings, three-dimensional HH-OCT analysis has the potential to change the way in which infants with optic nerve pathologies are managed, by providing detailed insight into disease progression.

**Table 5.** Recommended Protocol for Leica HH-OCT System (Envisu C2300) for Pediatric Imaging

	
Scan protocol	Use 12 mm x 8 mm, 600 A scans x 80 B scans. This rapid protocol is suitable for pediatric imaging, whilst providing sufficient image resolution.
‘Free-run’ mode	The use of the ‘free-run’ mode allows continuous real-time imaging of the optic nerve until the desired volume is viewed and captured.
En face	The use of retinal vessels to ensure no motion artefacts.
Distance	6° ≈ 1.73 mm radius, from the optic nerve center.
Visual angles	Defining lateral distances for RNFL thickness measures as visual angles eliminates the need for correction of axial length.
Reliability	Inferior and superior quadrants are suggested for reliability purposes.

## Supplementary Material

Supplement 1

Supplement 2

Supplement 3

Supplement 4
