# Supplementary material for: Reliability and Recommended Settings for Pediatric Circumpapillary Retinal Nerve Fiber Layer Imaging Using Hand-Held Optical Coherence Tomography
Source: Transl Vis Sci Technol. 2020 Jun 30;9(7):43. doi: 10.1167/tvst.9.7.43 (PMC7414610; doi:10.1167/tvst.9.7.43)
Supplement: Supplement 1 [file tvst-9-7-43_s001.pdf]

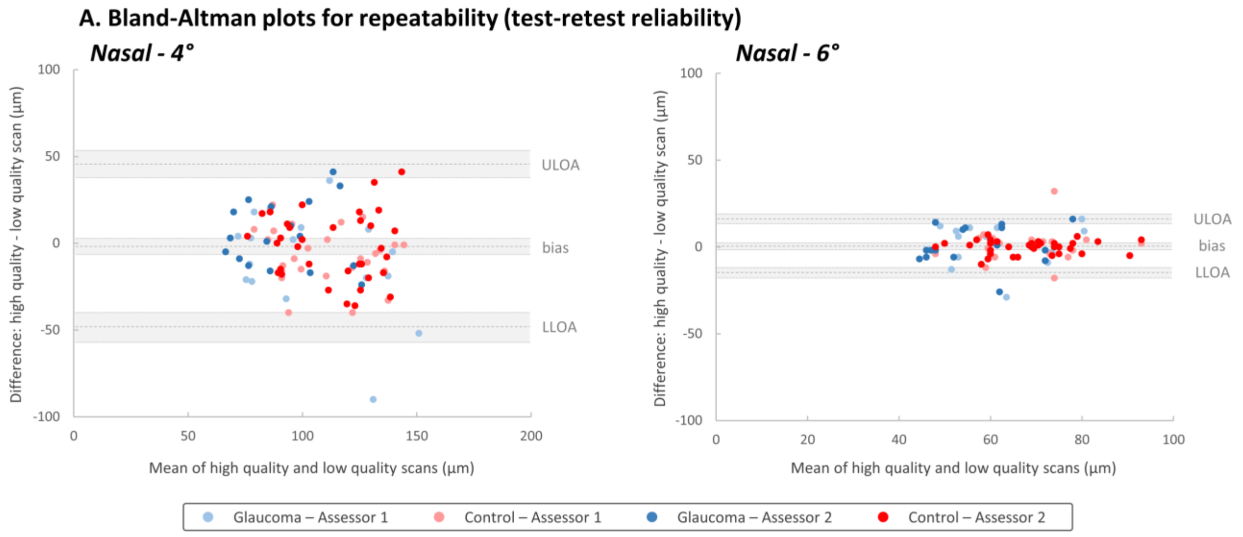

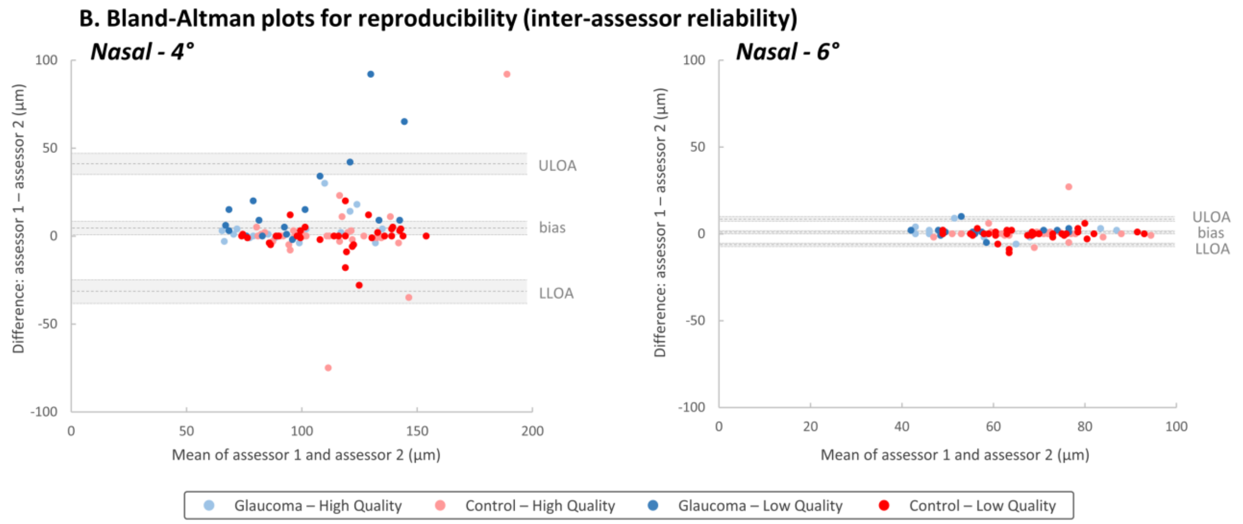

Supplementary Figure S1: Bland-Altman plots – Comparison between 4° to 6° from optic nerve center

Bland-Altman plots to illustrate the differences in mean RNFL thickness. The mean (dashed line) with 95% upper and lower confidence intervals (solid lines) are provided. (A) shows the improvement in reliability from 4° to 6° in the nasal quadrant, between high and low quality scans, and (B) between assessors.
