# Supplementary material for: Reliability and Recommended Settings for Pediatric Circumpapillary Retinal Nerve Fiber Layer Imaging Using Hand-Held Optical Coherence Tomography
Source: Transl Vis Sci Technol. 2020 Jun 30;9(7):43. doi: 10.1167/tvst.9.7.43 (PMC7414610; doi:10.1167/tvst.9.7.43)
Supplement: Supplement 2 [file tvst-9-7-43_s002.pdf]

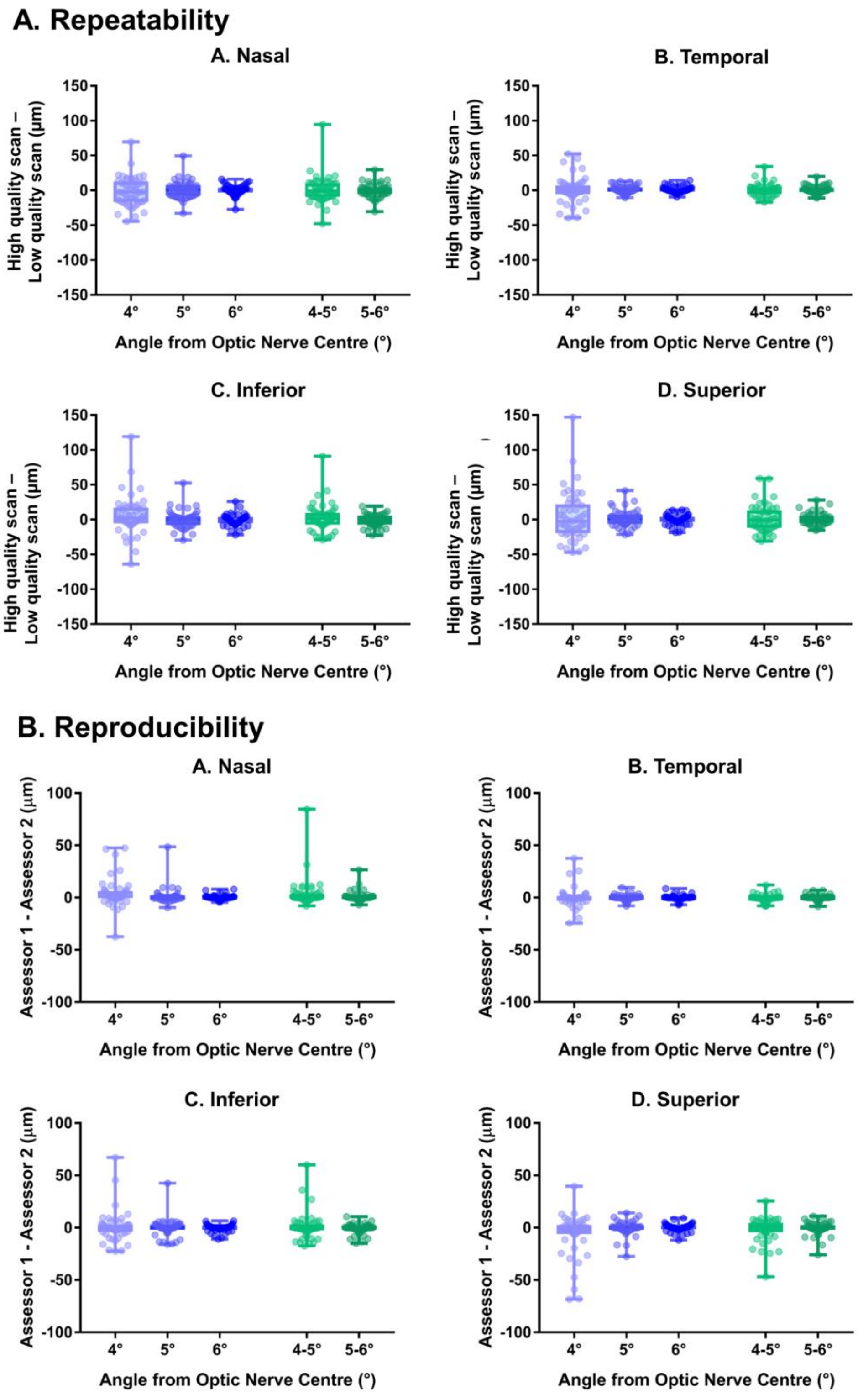

Supplementary Figure S2: Box and whisker plots, with individual data points, to represent difference values for (A) repeatability and (B) reproducibility.
